# Efficacy and Safety of Talc Pleurodesis for Malignant Pleural Effusion: A Meta-Analysis

**DOI:** 10.1371/journal.pone.0087060

**Published:** 2014-01-27

**Authors:** Huan Xia, Xiao-Juan Wang, Qiong Zhou, Huan-Zhong Shi, Zhao-Hui Tong

**Affiliations:** 1 Department of Respiratory and Critical Care Medicine, Beijing Chaoyang Hospital, Beijing Institute of Respiratory Medicine, Capital Medical University, Beijing, China; 2 Department of Respiratory and Critical Care Medicine, Union Hospital, Tongji Medical College, Huazhong University of Science and Technology, Wuhan, China; 3 Center of Medical Research, Beijing Chaoyang Hospital, Capital Medical University, Beijing, China; Clinica Universidad de Navarra, Spain

## Abstract

**Background:**

Talc pleurodesis has been widely used to control malignant pleural effusion; however, it is still not clear whether talc pleurodesis is more effective than other local therapies. We performed a meta-analysis to evaluate the efficacy and safety of talc pleurodesis in the management of malignant pleural effusion.

**Methods:**

PubMed, Embase, and Web of Science were searched for English-language studies of clinical controlled trials comparing talc pleurodesis with control therapies until August 8, 2013. Success rate and incidence of adverse events were evaluated. Relative risks were estimated using random- or fixed- effects model and statistical heterogeneity was assessed using *I^2^* test.

**Results:**

Twenty trials involving 1,525 patients with malignant pleural effusion were included. The success rate of talc pleurodesis was significantly higher than that of control therapies (relative risk, 1.21; 95% confidence interval, 1.01–1.45; p = 0.035) with similar adverse events. In addition, thoracoscopic talc poudrage was more effective than bedside talc slurry (relative risk, 1.12; 95% confidence interval, 1.01–1.23; p = 0.026).

**Conclusions:**

The current evidences suggested the benefit for talc pleurodesis in the treatment of malignant pleural effusion. Talc pleurodesis, especially thoracoscopic talc poudrage pleurodesis, should be performed in patients with malignant pleural effusion, especially those with life-expectancy longer than one month.

## Introduction

Malignant pleural effusion (MPE) is a common complication of advanced malignancy with a poor prognosis. It’s estimated that MPE affects more than 150,000 people each year in the United States [1]. Progressive dyspnea is the most common symptom in patients with MPE followed by cough and chest pain that affect the quality of life [2]. Although some malignancies such as small cell lung cancer, lymphoma, or breast cancer might respond to systemic treatment, local therapy for MPE may still be needed [3]. Local palliative procedures are more required to relieve dyspnea, improve life quality, and avoid repeated thoracentesis for patients not responding to systemic treatment [4]. Current local managements include thoracentesis, pleurodesis, chest tube drainage, indwelling pleural catheters drainage, pleurectomy, and pleuroperitoneal shunting [1], [5].

Pleurodesis is a procedure aiming at the adhesion of the visceral and parietal pleura that prevents the accumulation of MPE and subsequently improves symptoms [6]. Pleurodesis can be completed by chemical sclerosants or by physical abrasion of pleural surfaces during thoracoscopy or thoracotomy [6]. Pleurodesis after intercostal drainage is recommended for MPE management unless the lung is significantly trapped according to the current British Thoracic Society guideline [5].

Among the wide variety of sclerosing agents used to produce pleurodesis, talc is considered the most effective [1], [5]. A Cochrane review demonstrated that talc was the most effective sclerosant comparing with other sclerosants and a significant reduction in MPE recurrence [7]. Another systemic review revealed that talc tended to be associated with fewer recurrence of MPE when compared with other sclerosants, but there was no significant difference [8]. On the other hand, a few complications of talc pleurodesis existed, such as acute respiratory failure, pneumonia, and treat-related death [9], [10]. Actually, there is still controversy about the use of talc pleurodesis in clinical practice [11], [12]. We thus performed the current meta-analysis based on published controlled clinical trials to evaluate the overall efficacy and safety of talc pleurodesis compared with the other local therapies in patients with MPE.

## Methods

### Literature Search

PubMed, Embase, and Web of Science were searched for suitable studies up to August 8, 2013; no lower date limit was employed. Search keywords included: “malignant pleural effusion”, “pleurodesis”, and “talc”. Articles were also identified by use of the related-articles function in PubMed. References of articles identified were also searched manually. Although no language restrictions were imposed initially, for the full-text review and final analysis our resources only permitted review of English articles.

### Study Eligibility

We included full-text publications that investigated MPE patients treated with talc pleurodesis compared with other sclerosants or other local palliative methods, as well as those compared different routes of talc administration (talc poudrage *versus* talc slurry). We only selected randomized controlled trials and prospective unrandomized trials. Retrospective studies, case studies, letter reports, and conference abstracts were excluded. Publications with evidence of possible overlap of patients with other studies were discussed by HX, XJW, and QZ and only the best-quality study was used. Two reviewers (HZS and ZHT) independently judged study eligibility while screening the citations. Disagreements were resolved by consensus.

### Data Extraction and Quality Assessment

The final set of the English articles was assessed independently by two reviewers (HX and XJW). The following information was obtained from each trial: publication details (title, the first author, other citation details), study design, participants’ details (the numbers of patients enrolled, participant demographics, cancer type), the intervention details, the duration of follow-up, primary outcome (success rate) and adverse events. All data were verified for internal consistency, and discrepancy was resolved by discussion among the reviewers.

In the present meta-analysis, complete response (no accumulation of pleural effusion on the basis of radiographic evidence till the end of follow-up or death) was defined as successful pleurodesis. However, a few studies did not use this assessment criterion. For these studies, partial response (re-accumulation of pleural effusion but not requiring further therapeutic thoracentesis till the end of follow-up or death) was also defined as successful pleurodesis.

Quality assessment of the included studies was done unblindly by 2 reviewers (QZ and HZS) using a 10 point scoring system [13].

### Statistical Analysis

Relative risk (RR) with 95% confidence intervals (CI) was calculated using fixed- or random-effects model. Statistics heterogeneity was assessed by *I^2^* test with a value greater than 50% was recognized as indicative of substantial heterogeneity [14]. If substantial heterogeneity was observed, random-effects models were used. Meta-regression was performed to find the source of heterogeneity. Funnel plot was applied to assess the publication bias visually and Egger’s test was used to evaluate publication bias statistically [15]. A p value <0.05 was considered statistically significant. STATA 11.0 software was used in all analyses (Stata Corporation, College Station, TX).

## Results

### Eligible Trials

**Figure 1:**
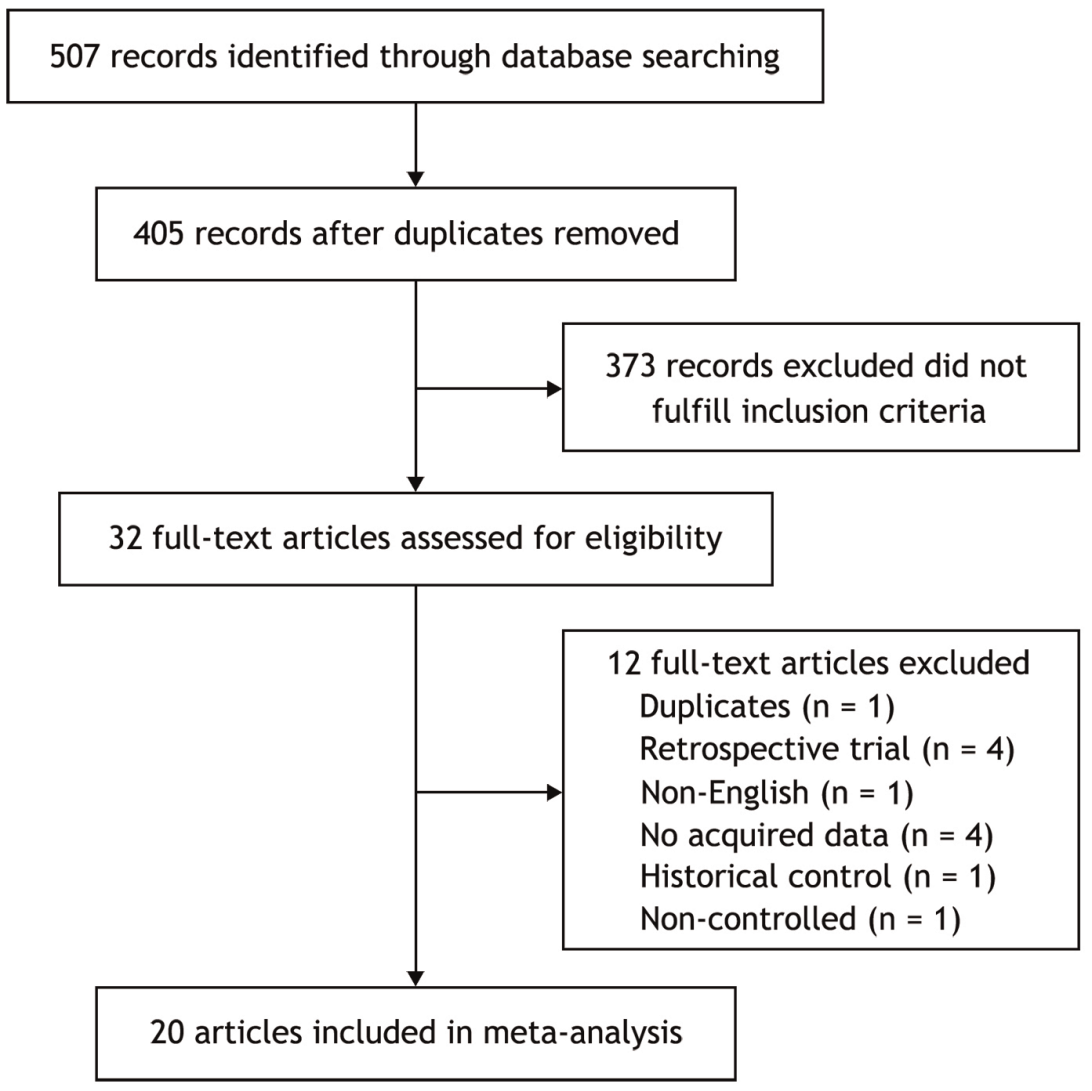
A flow chart showing the progress of trials through the review.

After an initial independent search, 507 potentially relevant publications were identified and then 102 duplicates were excluded. Titles and abstracts were reviewed and 373 publications were excluded which included animal experiments, retrospective studies, non-relevant papers, and non-controlled trials. Among the remaining 32 full-text articles, 1 was excluded because the same authors published 2 reports on the same patients and only the best-quality study was considered [16], 4 were excluded because they were retrospective studies [17]–[20], 1 were excluded because they were not English publications [21], 4 were excluded because they did not provide acquired data [22]–[25], 1 was excluded because it was historical controlled trial [26], 1 was excluded because it was not a controlled trial [27]. Subsequently, 20 articles were available for analyzing the efficacy or/and safety of talc pleurodesis for MPE [28]–[47] (Figure 1), and all the articles were the reports of randomized controlled trials except for two [42], [43].

### Study Characteristics and Quality

Twenty clinical trials involving 1,525 adult MPE patients were qualified for inclusion [28]–[47], and baseline characteristics of these 20 trials are listed in Table 1. Among these trials, 10 were conducted in Europe [28]–[31], [35], [37], [38], [42], [45], [46], 7 in America [32], [34], [39]–[41], [44], [47], and 3 in Asia [33], [36], [43]. Among 16 trials comparing talc pleurodesis with other therapies, 5 compared talc with bleomycin [31], [34]–[36], [39], 2 compared talc with povidone iodine [43], [45], 2 compared talc with indwelling pleural catheters [46], [47], 1 compared talc with bleomycin plus doxycycline [32], mustine [28], tetracycline [30], doxycycline [37], thoracoscopic mechanical pleurodesis [38], silver nitrate [41], and drainage alone [29], respectively. Four trials compared thoracoscopic talc poudrage with bedside talc slurry [33], [40], [42], [44].

**Table 1:** Characteristics and quality scores of the included studies.

Author/Year	StudyDesign	Regions	Cancer Types	TalcMethods	ControlTherapies	Follow-up	Quality Scores
Fentiman/1983	RCT	UK	Breast cancer	Poudrage	Mustine	1 m, then 3 monthly, at least 6 m	7
Sorensen/1984	RCT	Denmark	All cancer types	Slurry	Drainage	1 m, then 3 monthly	7
Fentiman/1986	RCT	UK	Breast cancer	Poudrage	Tetracycline	1 m, at least 12 m	7
Hamed/1989	RCT	UK	Breast cancer	Poudrage	Bleomycin	Median follow-up 24 m	5
Lynch/1996	RCT	USA	All cancer types	Slurry	Bleomycin,tetracycline	1 m	7
Yim/1996	RCT	Hong Kong	All cancer types	Poudrage	Talc slurry	6-wk intervals for the first 4^1^/_2_ mand then every 3 m	7
Zimmer/1997	RCT	USA	All cancer types	Slurry	Bleomycin	2 wk to 8 m	6
Diacon/2000	RCT	Swizerland	All cancer types	Poudrage	Bleomycin	1, 3 and 6 m	7
Ong/2000	RCT	Singapore	All cancer types	Slurry	Bleomycin	1 m	7
Kuzdzal/2003	RCT	Poland	All cancer types	Poudrage	Doxycycline	Until death or end of the study,at least 12 m	7
Haddad/2004	RCT	Brazil	All cancer types	Slurry	Bleomycin	1, 2 and 6 m	6
Crnjac/2004	RCT	Slovenia	All cancer types	Slurry	TMP	1, 3 and 6 m	7
Paschoalini/2005	RCT	Brazil	All cancer types	Slurry	Silver nitrate	1, 2, 3 and 4 m	8
Dresler/2005	RCT	USA	All cancer types	Poudrage	Talc slurry	1 m	7
Stefani/2006	Non-RCT	Italy	All cancer types	Poudrage	Talc slurry	1, 3 m and end of follow-up	5
Das/2008	Non-RCT	India	All cancer types	Slurry	Povidone iodine	6 m	5
Terra/2009	RCT	Brazil	All cancer types	Poudrage	Talc slurry	1, 3 m and every 3 m	7
Mohsen/2011	RCT	UK	Breast cancer	Poudrage	Povidone iodine	Every 3 m	8
Davies/2012	RCT	UK	All cancer types	Slurry	IPC	12 m	7
Demmy/2012	RCT	USA	All cancer types	Slurry	IPC	1 m	7

RCT = randomized controlled trial; TMP = thoracoscopic mechanical pleurodesis; IPC = indwelling pleural catheter.

The mean quality score was 6.7, with a range from 5 to 8 (Table 1). Therefore, the overall quality of all trials was not very good, especially none was blind designed.

### Overall Efficacy

**Figure 2:**
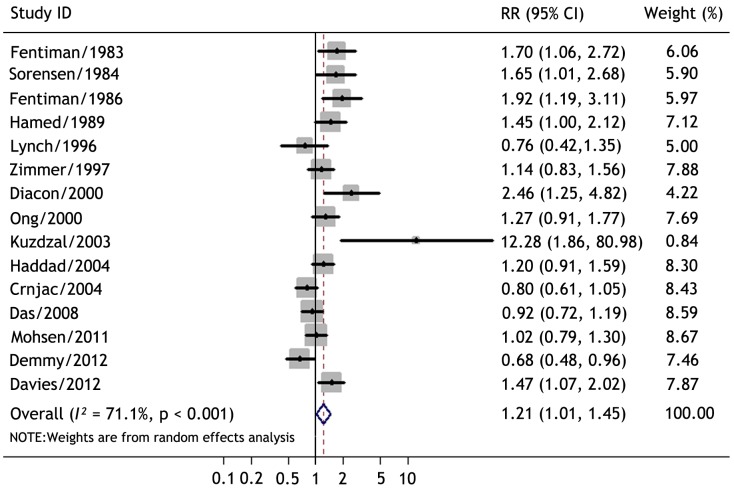
Meta-analysis of the relative risks (RR) of success rate between talc pleurodesis and control therapies using random-effects model. Bars represent 95% confidence intervals (CI) of RR in patients receiving talc pleurodesis versus controls. The center of the lozenge gives the combined RR. RR was considered statistically significant if the 95% CI for the overall RR does not overlap one.

Talc was compared with other sclerosants (mustine, tetracycline, bleomycin, doxycycline, sliver nitrate, povidone iodine) and other therapeutic regimen (indwelling pleural catheters, thoracoscopic mechanical pleurodesis, and drainage alone) either by thoracoscopic poudrage or bedside slurry in 15 studies involving 684 patients [28]–[32], [34]–[39], [43], [45]–[47]. The overall efficacy was evaluated according to the longest follow-up time in each trial. The study by Paschoalini et al was excluded from this evaluation because only 4 patients were available for the longest follow-up [41]. Overall RR of success rate favored talc pleurodesis compared with control therapies (RR, 1.21; 95% CI, 1.01–1.45; p = 0.035; random-effects model) (Figure 2).

It should be noted that the follow-up time of assessing outcome varied from 1 m to several months. Seven studies [32], [35], [36], [38], [39], [41], [47] reported success rate of talc pleurodesis compared with control therapies at 1 m, while 11 studies [28]–[31], [35], [37]–[39], [43], [45], [46] at longer than 1 m. Our meta-analysis revealed that no significant difference was observed at 1 m (RR, 0.94; 95% CI, 0.80–1.11; p = 0.455; random-effects model); in contrast, talc pleurodesis was more effective than control therapies (RR, 1.35; 95% CI, 1.07–1.69; p = 0.010; random-effects model) at longer than 1 m (Table 2).

**Table 2:** Comparision of success rate between talc pleurodesis and control therapies at 1 month and longer than 1 month.

Assessing Time	Study/Year	Talc Pleurodesis(n/N)	Control Therapies(n/N)	RR (95% CI)	*I^2^*, P (het)	P (Z)
1 month						
	Lynch/1996	8/17	18/29	0.76 (0.42–1.35)		
	Diacon/2000	13/15	10/17	1.47 (0.94–2.30)		
	Ong/2000	16/18	14/20	1.27 (0.91–1.77)		
	Crnjac/2004	27/38	40/45	0.80 (0.64–1.00)		
	Haddad/2004	33/37	30/34	1.01 (0.86–1.19)		
	Paschoalini/2005	21/25	23/24	0.88 (0.72–1.06)		
	Demmy/2012	15/25	23/26	0.68 (0.48–0.96)		
	Overall	133/175	158/195	0.94 (0.80–1.11)	58.1%, 0.026	0.455
Longer than 1 month						
	Fentiman/1983	18/20	9/17	1.70 (1.06–2.72)		
	Sorensen/1984	9/9	7/12	1.65 (1.01–2.68)		
	Fentiman/1986	11/12	10/21	1.92 (1.19–3.11)		
	Hamed/1989	10/10	10/15	1.45 (1.00–2.12)		
	Diacon/2000	13/15	6/17	2.46 (1.25–4.82)		
	Kuzdzal/2003	17/18	1/13	12.28 (1.86–80.98)		
	Haddad/2004	30/37	23/34	1.20 (0.91–1.59)		
	Crnjac/2004	23/34	33/39	0.80 (0.61–1.05)		
	Das/2008	19/24	24/28	0.92 (0.72–1.19)		
	Mohsen/2011	19/22	17/20	1.02(0.79–1.30)		
	Davis/2012	36/48	26/51	1.47 (1.07–2.02)		
	Overall	205/249	166/267	1.35 (1.07 − 1.69)	74.9%, <0.001	0.010

n = the number of patients with successful pleurodesis; N = the number of evaluable patients for outcome; RR = relative risk;

CI = confidence interval.

**Figure 3:**
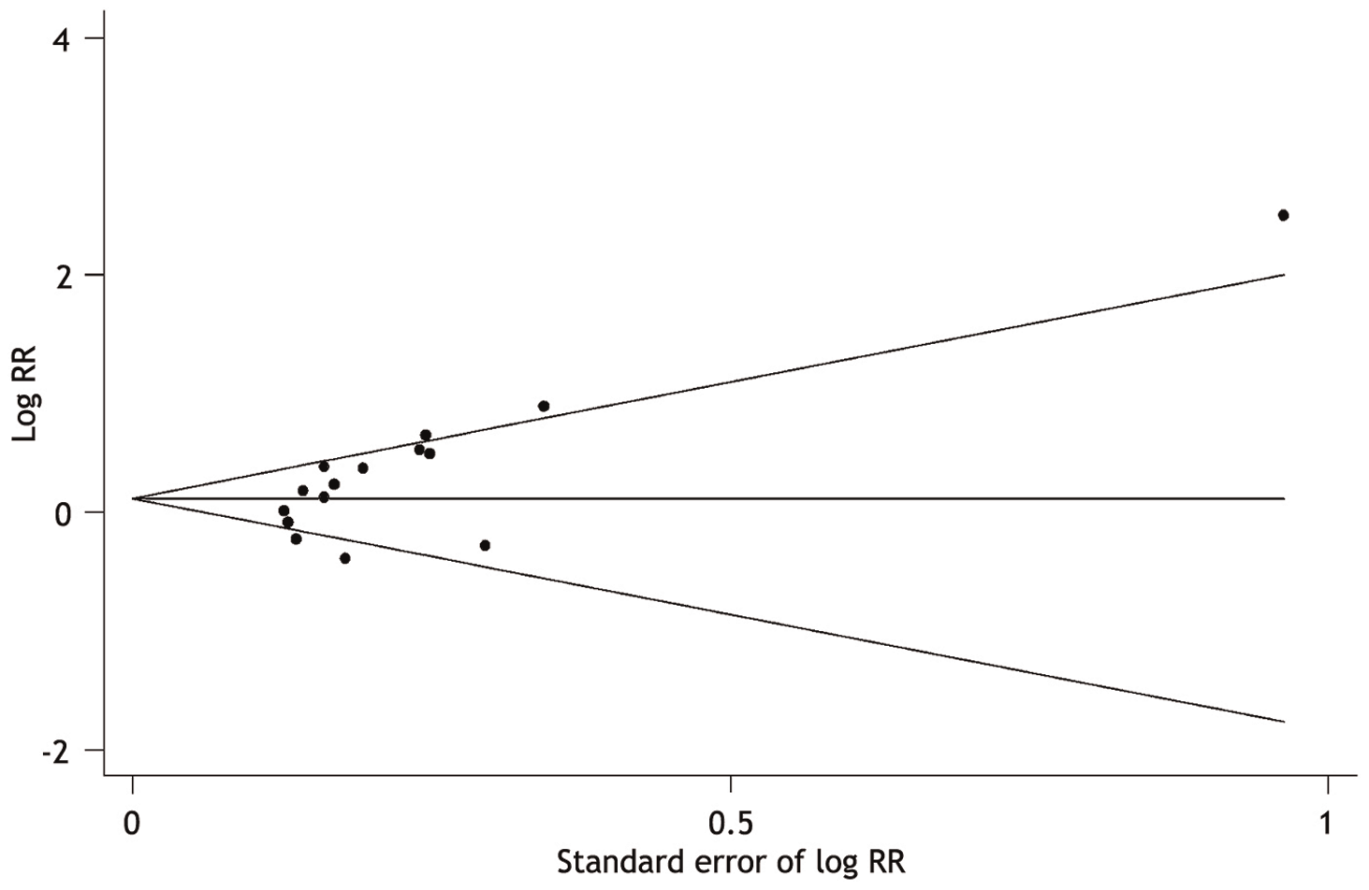
Funnel graph for the assessment of potential publication bias in talc pleurodesis compared with control therapies for malignant pleural effusion. The funnel graph plots log of relative risk (RR) against the standard error of the log of RR. Each solid circle represents each study in the meta-analysis. The line in the center indicates the summary RR.

Overall test for heterogeneity showed that *I^2^* = 71.1% (p<0.001), indicating a significant heterogeneity between studies. The funnel plots for publication bias showed some asymmetry (Figure 3), and the Egger’s test was significant (p = 0.015), indicating there was a publication bias.

Due to the significant heterogeneity between studies in assessing the overall efficacy of talc pleurodesis, we performed a meta-regression to explore the source of the heterogeneity. It was noted that the routes of administration, control therapies, methods of assessing success rate, and cancer types were not the source of heterogeneity (all p>0.05).

### Subgroup Analysis

Four studies involving 508 patients compared the efficacy of thoracoscopic talc poudrage with bedside talc slurry [33], [40], [42], [44]. Our meta-analysis indicated that thoracoscopic talc poudrage was more effective (RR, 1.12; 95% CI, 1.01–1.23; p = 0.026; fixed-effects model) (Table 3).

**Table 3:** Subgroup analysis of success rates between talc pleurodesis and different control groups.

Comparison Groups	Study	Talc pleurodesis(n/N)	Control Therapies(n/N)	RR (95% CI)	*I^2^*, P (het)	P (Z)
Talc poudrage *vs* Talc slurry						
	Yim/1996	27/28	26/29	1.08 (0.93–1.24)		
	Dresler/2005	119/152	92/130	1.11 (0.96–1.27)		
	Stefani/2006	59/72	23/37	1.32 (1.00–1.73)		
	Terra/2009	25/30	26/30	0.96 (0.78–1.19)		
	Overall	230/282	167/226	1.12 (1.01–1.23)	16.5%, 0.309	0.026
Talc poudrage *vs* controls						
	Fentiman/1983	18/20	9/17	1.70 (1.06–2.72)		
	Fentiman/1986	11/12	10/21	1.92 (1.19–3.11)		
	Hamed/1989	10/10	10/15	1.45 (1.00–2.12)		
	Diacon/2000	13/15	6/17	2.46 (1.25–4.82)		
	Kuzdzal/2003	17/18	1/13	12.28 (1.86–80.98)		
	Mohsen/2011	19/22	17/20	1.02 (0.79–1.30)		
	Overall	88/97	53/103	1.74 (1.11–2.73)	80.5%, <0.001	0.015
Talc slurry *vs* controls						
	Sorensen/1984	9/9	7/12	1.65 (1.01–2.68)		
	Lynch/1996	8/17	18/29	0.76 (0.42–1.35)		
	Zimmer/1997	17/19	11/14	1.14 (0.83–1.56)		
	Ong/2000	16/18	14/20	1.27 (0.91–1.77)		
	Haddad/2004	30/37	23/34	1.20 (0.91–1.59)		
	Crnjac/2004	23/34	33/39	0.80 (0.61–1.05)		
	Das/2008	19/24	24/28	0.92 (0.72–1.19)		
	Demmy/2012	15/25	23/26	0.68 (0.48–0.96)		
	Davis/2012	36/48	26/51	1.47 (1.07–2.02)		
	Overall	173/231	179/253	1.05 (0.87–1.27)	64.0%, 0.004	0.588
Talc *vs* Bleomycin						
	Hamed/1989	10/10	10/15	1.45 (1.00–2.12)		
	Lynch/1996	8/17	10/14	0.66 (0.36–1.20)		
	Zimmer/1997	17/19	11/14	1.14 (0.83–1.56)		
	Diacon/2000	13/15	6/17	2.46 (1.25–4.82)		
	Ong/2000	16/18	14/20	1.27 (0.91–1.77)		
	Haddad/2004	30/37	23/34	1.20 (0.91–1.59)		
	Overall	94/116	74/114	1.25 (1.06–1.46)	45.8%, 0.100	0.007
Talc *vs* Tetracycline						
	Fentiman/1986	11/12	10/21	1.92 (1.19–3.11)		
	Lynch/1996	8/17	8/15	0.88 (0.44–1.76)		
	Overall	19/29	18/36	1.36 (0.62–2.97)	71.5%, 0.061	0.448
Talc *vs* Povidone iodine						
	Das/2008	19/24	24/28	0.92 (0.72–1.19)		
	Mohsen/2011	19/22	17/20	1.02 (0.79–1.30)		
	Overall	38/46	41/48	0.97 (0.81–1.15)	0.0%, 0.597	0.695
Talc *vs* IPC						
	Demmy/2012	15/25	23/26	0.68 (0.48–0.96)		
	Davis/2012	36/48	26/51	1.47 (1.07–2.02)		
	Overall	51/73	49/77	1.00 (0.47–2.16)	90.6%, 0.001	0.995
Talc *vs* Others*						
	Fentiman/1983	18/20	9/17	1.70 (1.06–2.72)		
	Sorensen/1984	9/9	7/12	1.65 (1.01–2.68)		
	Kuzdzal/2003	17/18	1/13	12.28 (1.86–80.98)		
	Crnjac/2004	23/34	33/39	0.80 (0.61–1.05)		
	Overall	67/81	50/81	1.61 (0.79–3.27)	86.4%, <0.001	0.187

RR = relative risk; n = the number of patients with successful pleurodesis; N = the number of evaluable patients for outcome; IPC = indwelling pleural catheter.

* Others included mustine, silver nitrate, doxycycline, thoracoscopic mechanical pleurodesis, and drainage alone.

When compared with control therapies, talc was administered either by thoracoscopic poudrage [28], [30], [31], [35], [37], [45] or by bedside slurry [29], [32], [34], [36], [38], [39], [43], [46], [47]. We performed a subgroup analysis by routes of administration separately, and found that thoracoscopic talc poudrage was much superior to control therapies in controlling MPE compared with other local therapies (RR, 1.74; 95% CI, 1.11–2.73; p = 0.015) (Table 3). Whereas there was no significant difference in efficacy between talc slurry and controls (RR, 1.05, 95% CI, 0.87–1.27; p = 0.588).

We also performed a meta-analysis of efficacy of talc pleurodesis stratified by different controls, including bleomycin, tetracycline, povidone, indwelling pleural catheters, and the others (mustine, silver nitrate, drainage alone, doxycycline, and thoracoscopic mechanical pleurodesis, etc.) (Table 3). Compared with bleomycin pleurodesis, talc pleurodesis was more effective (RR, 1.25; 95% CI, 1.06–1.46; p = 0.007). The efficacy of talc pleurodesis was similar to any other control arms (all p>0.05).

Four studies [28], [30], [31], [45] chose patients with MPE secondary to carcinoma of the breast as the study population and the other 11 studies [29], [32], [34]–[39], [43], [46], [47] included patients with MPE in spite of the types of malignancies. As a result, we did an analysis according to whether the patients were all with breast cancer or not. The results suggest that talc pleurodesis was more effective than control therapies in MPE patients with breast cancer (RR 1.42; 95% CI, 1.02–1.46; p = 0.035). The efficacy was similar in the group without limitation of cancer types (RR 1.43; 95% CI, 0.92–1.42; p = 0.233).

### Adverse Events

Six studies reported the incidence of fever [32], [35], [36], [41], [43], [45], and the meta-analysis did not identify significant difference between talc pleurodesis and control therapies (RR, 1.15; 95% CI, 0.69–1.94; p = 0.589; fixed-effects model).

Six studies reported the incidence of pain [29], [32], [36], [43], [45], [47]. There was no significant difference between talc pleurodesis and control therapies (RR, 0.74; 95% CI, 0.40–1.40; p = 0.360; fixed-effects model).

Data on emphysema events was reported in 4 studies [28], [30], [38], [46]. Our result showed no significant difference between talc pleurodesis and control therapies (RR, 1.35; 95% CI, 0.45–4.08; p = 0.596; fixed-effects model).

Five studies reported the incidence of wound infection [28], [30], [34], [38], [47]. There was no significant difference between talc pleurodesis and control therapies (RR, 2.18; 95% CI, 0.85–5.58; p = 0.106; fixed-effects model).

## Discussion

Once a patient is diagnosed as MPE, his median survival is just 4 to 6 m depending on the type of malignancy [1]. Most patients with MPE are symptomatic and dyspnea is the most common symptom affecting the quality of life [2]. Therapeutic thoracentesis is effective in alleviating symptom, but MPE usually recur within 1 m [48], [49]. Furthermore, repeated thoracentesis has the risk of pneumothorax, empyema and pleural adhesions which influence the following procedure such as drainage and thoracoscopy [5].

Talc pleurodesis results in the symphysis between the visceral and parietal pleura that prevents the accumulation of liquid in the pleural space, and usually has the advantage of a time-limited course of treatment and high pleurodesis rate. Moreover, talc has been demonstrated to possess a local antitumor effect of by triggering apoptosis in cancer cells [50] and by altering the angiostatic balance via endostatin [51], suggesting that talc might play a significant role in controlling not only MPE but also intrapleural tumor progression. In the present meta-analysis, we found that talc pleurodesis was associated with higher success rates compared with control therapies, and that thoracoscopic talc poudrage was more effective in controlling MPE than bedside talc slurry pleurodesis.

The assessing time of success rate of talc pleurodesis in the included studies varied from 1 m to several months or until death, we therefore evaluated the short-term (1 m) and long-term (longer than 1 m) efficacy separately. We noted that the efficacy of talc pleurodesis compared with control therapies at 1 m was similar; whereas the efficacy evaluated at longer than 1 m favored talc pleurodesis other than the controls. These data indicated that MPE patients with life expectancy more than 1 m would benefit more from talc pleurodesis.

Although talc pleurodesis is now well recognized as the procedure of choice for the treatment of MPE, the optimal route of talc administration is still debated. Some authors prefer thoracoscopic talc poudrage, but the others advocate talc instillation through a chest tube because it was a simpler and less invasive procedure. Our subgroup analysis demonstrated that thoracoscopic talc poudrage was superior in managing MPE to control therapies, while the success rates of talc slurry and control therapies were not significantly different. Combining with the above results of comparing thoracoscopic talc poudrage and talc slurry, thoracoscopic talc poudrage could be regarded as the better choice of MPE management. It is reasonable that slurry procedure is not favorable because it does not allow the talc to be thoroughly distributed over the whole pleural surface [52].

In the overall analysis, the control therapies included chemical sclerosants and other local therapies such as indwelling pleural catheters, thoracoscopic mechanical pleurodesis, and drainage alone. Therefore, we divided the control groups into five arms and performed subgroup analysis: bleomycin, tetracycline, povidone iodine, indwelling pleural catheters, and the others. Our results supported that the RR of successful pleurodesis favors talc pleurodesis other than bleomycin pleurodesis, but not any other control therapies. Among total 6 studies comparing talc and bleomycin pleurodesis, talc poudrage was used in 2 studies [31], [35] and talc slurry was used in the remaining 4 studies [32], [34], [36], [39]. Further subgroup analysis revealed that the efficacy of talc poudrage (RR, 1.85; 95% CI, 1.28–2.68; p = 0.001), but not of talc slurry (RR, 1.10; 95% CI, 0.93–1.32; p = 0.276), was superior to that of bleomycin pleurodesis.

The tumor type involved in the pleural surfaces may influence the success rate of pleurodesis [6]. Therefore, we conducted an analysis according to different study population. In the 4 studies which only included MPE patients with breast cancer, talc pleurodesis was more effective (RR 1.42; 95% CI 1.02–1.46; p = 0.035). However, in 11 studies which included MPE patients with breast cancer or other cancers, the success rate was similar. These results might suggest the superiority of talc pleurodesis for patients with MPE secondary to breast cancer. But the present study is not an individual patient data meta-analysis and the data is small. Further controlled studies are needed to confirm the influence of tumor type on the outcome of talc pleurodesis.

The current British Thoracic Society guideline [5] advocated talc pleurodesis as first-line therapy for MPE, with indwelling pleural catheters reserved for second-line treatment or for those without complete lung reexpansion. Intermittent external drainage through indwelling pleural catheters is gaining popularity, because indwelling pleural catheters can be inserted as an outpatient procedure and offer rapid relief of dyspnea through ambulatory drainage of MPE [53], [54]. The results of this meta-analysis demonstrated that both talc pleurodesis and indwelling catheters were effective initial treatments for controlling MPE with similar success. There might be the other advantages to the use of indwelling pleural catheters, such as reduced hospital stay and decreased requirement for further pleural procedures [3], [52], this procedure could therefore be alternative to talc pleurodesis, especially talc slurry pleurodesis.

Although talc has been used as sclerosing agents for a long time, not everyone accepted talc pleurodesis for treating MPE because of its complications [10], [12]. Talc causes a severe chemical pleurisy resulting in effective pleurodesis, but can also worsen pain and dyspnea in these patients and can result in respiratory failure [1], [6]. Other complications associated with talc pleurodesis include fever, acute pneumonitis, and empyema. However, only a few side effects or complications were reported in the included studies. Based on the limited data, we found no significant difference in incidence of fever, pain, emphysema, and wound infection between talc pleurodesis and control therapies. Unfortunately, no required data could be obtained from the included studies for pooled analyzing the incidence of respiratory failure and the other complications. Totally, compared with control therapies, talc pleurodesis is safe and more effective in managing MPE.

Importantly, it should be kept in mind that the goal of local palliative treatment is not survival but the prevention of MPE re-accumulation and relief of symptoms in patients’ last months. When making choice in clinical practice, one should take into account the economic cost, patients’ performance status and their preference, etc.

Several limitations of this present meta-analysis should be mentioned. First, this meta-analysis was based on the published literature, not on individual patient data. Meta-analysis based on published data tends to overestimate treatment effects compared with individual patient data analysis. The results of our meta-analysis must therefore be interpreted cautiously, since an individual patient data-based meta-analysis would give more reliable estimation than one based on abstracted data [55]. Second, most studies were small and had limitations, including that none was blind designed, and the overall quality of all studies was not very good. On the other hand, the nature of the interventions used for MPE management means that blinding was not possible. Third, heterogeneity among trials may be another limitation of our meta-analysis, even though we applied a random-effects model that takes possible heterogeneity into consideration. Fourth, publication bias is a significant threat to the validity of the results, which indeed existed in the present meta-analysis. Exclusion of conference abstracts, letters to the editors and non-English language studies may have led to publication bias.

Since the remaining lifespan of patients with MPE is very limited, the goal of MPE treatment should be rapid and durable relief of symptoms. The ideal therapy should be simple, safe, well tolerated, of lifelong success, and with minimal recurrence rate. The current evidence supported a benefit for talc pleurodesis in the treatment of patients with MPE. Thoracoscopic talc pleurodesis was highly effective and clearly superior to the other control therapies, especially bleomycin instillation, and especially for a longer term management of MPE.

## Supporting Information

Checklist S1 A PRISMA checklist for this meta-analysis.
(DOC)
